# Supplementary material for: Single Cell Transcriptomic and Chromatin Profiles Suggest Layer Vb Is the Only Layer With Shared Excitatory Cell Types in the Medial and Lateral Entorhinal Cortex
Source: Front Neural Circuits. 2022 Jan 26;15:806154. doi: 10.3389/fncir.2021.806154 (PMC8826650; doi:10.3389/fncir.2021.806154)
Supplement: Supplementary file 14 [file Data_Sheet_1.ZIP › 3/knownResults.html]

3 - Homer Known Motif Enrichment Results


# Homer Known Motif Enrichment Results (3)

Homer *de novo* Motif Results  
Gene Ontology Enrichment Results  
Known Motif Enrichment Results (txt file)  
Total Target Sequences = 1000, Total Background Sequences = 48169

|  |  |  |  |  |  |  |  |  |  |  |  |
| --- | --- | --- | --- | --- | --- | --- | --- | --- | --- | --- | --- |
| Rank | Motif | Name | P-value | log P-pvalue | q-value (Benjamini) | # Target Sequences with Motif | % of Targets Sequences with Motif | # Background Sequences with Motif | % of Background Sequences with Motif | Motif File | SVG |
| 1 | A G C T G T C A C G T A A C G T A C G T C T G A T C A G A T G C | Lhx2(Homeobox)/HFSC-Lhx2-ChIP-Seq(GSE48068)/Homer | 1e-146 | -3.368e+02 | 0.0000 | 636.0 | 63.60% | 11979.7 | 24.87% | motif file (matrix) | svg |
| 2 | A T G C T A C G A G T C G A C T T G C A C T G A G A C T A C G T C T G A T C A G | LHX9(Homeobox)/Hct116-LHX9.V5-ChIP-Seq(GSE116822)/Homer | 1e-125 | -2.889e+02 | 0.0000 | 673.0 | 67.30% | 14706.6 | 30.53% | motif file (matrix) | svg |
| 3 | A T C G A T C G A T G C G A C T T C G A C G T A G C A T A G C T C T G A T A C G | DLX2(Homeobox)/BasalGanglia-Dlx2-ChIP-seq(GSE124936)/Homer | 1e-117 | -2.698e+02 | 0.0000 | 705.0 | 70.50% | 16681.3 | 34.63% | motif file (matrix) | svg |
| 4 | A T G C T A C G A G C T T G C A C G T A C G A T A C G T C T G A | DLX5(Homeobox)/BasalGanglia-Dlx5-ChIP-seq(GSE124936)/Homer | 1e-116 | -2.686e+02 | 0.0000 | 514.0 | 51.40% | 9108.7 | 18.91% | motif file (matrix) | svg |
| 5 | C G T A C A T G A G T C G A C T T G C A C G T A A C G T A C G T C T G A T C A G | Lhx1(Homeobox)/EmbryoCarcinoma-Lhx1-ChIP-Seq(GSE70957)/Homer | 1e-114 | -2.627e+02 | 0.0000 | 625.0 | 62.50% | 13427.9 | 27.88% | motif file (matrix) | svg |
| 6 | T C A G C T A G A T G C G A C T T G C A C G T A A G C T A C G T C T G A T A C G | En1(Homeobox)/SUM149-EN1-ChIP-Seq(GSE120957)/Homer | 1e-110 | -2.537e+02 | 0.0000 | 735.0 | 73.50% | 18603.1 | 38.62% | motif file (matrix) | svg |
| 7 | A T C G A T G C A G T C T A G C G A C T T C G A G C T A G C A T A G C T C T G A | DLX1(Homeobox)/BasalGanglia-Dlx1-ChIP-seq(GSE124936)/Homer | 1e-109 | -2.511e+02 | 0.0000 | 649.0 | 64.90% | 14766.9 | 30.66% | motif file (matrix) | svg |
| 8 | T C G A C A G T A C T G A G C T C G T A C G T A A C G T A C G T C T G A T A G C | Dlx3(Homeobox)/Kerainocytes-Dlx3-ChIP-Seq(GSE89884)/Homer | 1e-108 | -2.491e+02 | 0.0000 | 473.0 | 47.30% | 8165.1 | 16.95% | motif file (matrix) | svg |
| 9 | C G T A C G T A A G C T G A C T T G C A G T C A A C G T A G C T C T G A T C A G | Lhx3(Homeobox)/Neuron-Lhx3-ChIP-Seq(GSE31456)/Homer | 1e-89 | -2.065e+02 | 0.0000 | 699.0 | 69.90% | 18493.4 | 38.39% | motif file (matrix) | svg |
| 10 | A C T G C A G T A C G T C G T A C G T A A C G T A C T G C T G A | Nkx6.1(Homeobox)/Islet-Nkx6.1-ChIP-Seq(GSE40975)/Homer | 1e-86 | -1.999e+02 | 0.0000 | 845.0 | 84.50% | 26528.4 | 55.08% | motif file (matrix) | svg |
| 11 | A G T C C G A T C T G A C G T A A C G T C A G T T C A G T G A C | Isl1(Homeobox)/Neuron-Isl1-ChIP-Seq(GSE31456)/Homer | 1e-67 | -1.561e+02 | 0.0000 | 644.0 | 64.40% | 17856.6 | 37.07% | motif file (matrix) | svg |
| 12 | C T G A T G A C T G A C C G T A A C G T T G A C A G C T C T A G A C G T G A C T | Olig2(bHLH)/Neuron-Olig2-ChIP-Seq(GSE30882)/Homer | 1e-60 | -1.391e+02 | 0.0000 | 531.0 | 53.10% | 13601.5 | 28.24% | motif file (matrix) | svg |
| 13 | T C G A T G A C G T A C C G T A C A G T T G A C A C G T A C T G A G C T A G C T | NeuroG2(bHLH)/Fibroblast-NeuroG2-ChIP-Seq(GSE75910)/Homer | 1e-41 | -9.612e+01 | 0.0000 | 387.0 | 38.70% | 9604.2 | 19.94% | motif file (matrix) | svg |
| 14 | T A C G T G C A A G T C C G T A A C G T T G A C A C G T A C T G A C T G G C A T | TCF4(bHLH)/SHSY5Y-TCF4-ChIP-Seq(GSE96915)/Homer | 1e-30 | -6.939e+01 | 0.0000 | 357.0 | 35.70% | 9636.0 | 20.01% | motif file (matrix) | svg |
| 15 | C T A G T C G A T G A C A G T C C G T A A C T G G T A C A C G T A C T G A C T G | BHLHA15(bHLH)/NIH3T3-BHLHB8.HA-ChIP-Seq(GSE119782)/Homer | 1e-29 | -6.897e+01 | 0.0000 | 324.0 | 32.40% | 8382.8 | 17.40% | motif file (matrix) | svg |
| 16 | T G A C A G T C C G T A A C T G G T A C A C G T A C T G A C G T G A C T G A T C | Twist2(bHLH)/Myoblast-Twist2.Ty1-ChIP-Seq(GSE127998)/Homer | 1e-28 | -6.656e+01 | 0.0000 | 387.0 | 38.70% | 10974.3 | 22.78% | motif file (matrix) | svg |
| 17 | T C A G T G A C G T A C C G T A A C G T T G A C A C G T T C A G A G C T G A C T | NeuroD1(bHLH)/Islet-NeuroD1-ChIP-Seq(GSE30298)/Homer | 1e-27 | -6.375e+01 | 0.0000 | 206.0 | 20.60% | 4388.4 | 9.11% | motif file (matrix) | svg |
| 18 | C T A G T A C G G A T C G T A C G C T A A G C T A G C T G T C A T C G A T A G C | Nanog(Homeobox)/mES-Nanog-ChIP-Seq(GSE11724)/Homer | 1e-25 | -5.801e+01 | 0.0000 | 806.0 | 80.60% | 31574.1 | 65.55% | motif file (matrix) | svg |
| 19 | T C A G A C G T T C G A T A G C A G T C C G T A A C T G G T A C A C G T A C T G A T C G A G T C | Atoh1(bHLH)/Cerebellum-Atoh1-ChIP-Seq(GSE22111)/Homer | 1e-22 | -5.091e+01 | 0.0000 | 231.0 | 23.10% | 5762.4 | 11.96% | motif file (matrix) | svg |
| 20 | C T G A T C A G G T A C G C T A A C T G T G A C G C A T C A T G | SCL(bHLH)/HPC7-Scl-ChIP-Seq(GSE13511)/Homer | 1e-14 | -3.419e+01 | 0.0000 | 645.0 | 64.50% | 25085.3 | 52.08% | motif file (matrix) | svg |
| 21 | T A C G A T C G G A T C G T C A C T G A G C A T C G A T G C T A T C G A G C T A | Unknown(Homeobox)/Limb-p300-ChIP-Seq/Homer | 1e-14 | -3.246e+01 | 0.0000 | 257.0 | 25.70% | 7768.4 | 16.13% | motif file (matrix) | svg |
| 22 | C T A G T A G C A G T C G T C A C T G A A C G T C G T A C G T A C G T A G C T A | Hoxd13(Homeobox)/ChickenMSG-Hoxd13.Flag-ChIP-Seq(GSE86088)/Homer | 1e-13 | -3.095e+01 | 0.0000 | 496.0 | 49.60% | 18256.4 | 37.90% | motif file (matrix) | svg |
| 23 | T C G A A C G T A C G T C T G A G A T C T C A G G A C T G T C A C G T A A G C T G T C A C T A G A G C T A C G T T C G A | NFIL3(bZIP)/HepG2-NFIL3-ChIP-Seq(Encode)/Homer | 1e-9 | -2.215e+01 | 0.0000 | 195.0 | 19.50% | 6022.8 | 12.50% | motif file (matrix) | svg |
| 24 | T C A G G A C T C A G T C T G A A G C T C T A G G A C T T G C A C T G A A G T C | HLF(bZIP)/HSC-HLF.Flag-ChIP-Seq(GSE69817)/Homer | 1e-8 | -2.033e+01 | 0.0000 | 250.0 | 25.00% | 8421.0 | 17.48% | motif file (matrix) | svg |
| 25 | G A C T C A G T A G C T C G A T A G T C G A T C A G T C C G T A A T G C T C A G | Rbpj1(?)/Panc1-Rbpj1-ChIP-Seq(GSE47459)/Homer | 1e-8 | -1.880e+01 | 0.0000 | 261.0 | 26.10% | 9028.9 | 18.75% | motif file (matrix) | svg |
| 26 | C G A T A C G T C G T A C G T A A C G T A G C T T G C A C T G A C G T A A C G T A C G T C G T A | Phox2b(Homeobox)/CLBGA-PHOX2B-ChIP-Seq(GSE90683)/Homer | 1e-7 | -1.708e+01 | 0.0000 | 104.0 | 10.40% | 2865.7 | 5.95% | motif file (matrix) | svg |
| 27 | C T A G T C G A A C G T A C T G C G T A A T G C A C G T G T A C C G T A A G C T G A T C G T A C | Atf3(bZIP)/GBM-ATF3-ChIP-Seq(GSE33912)/Homer | 1e-7 | -1.702e+01 | 0.0000 | 142.0 | 14.20% | 4312.8 | 8.95% | motif file (matrix) | svg |
| 28 | T C G A C T G A C T G A A T G C G T A C G T C A A G C T A G C T C G T A T C A G | Barx1(Homeobox)/Stomach-Barx1.3xFlag-ChIP-Seq(GSE69483)/Homer | 1e-7 | -1.700e+01 | 0.0000 | 182.0 | 18.20% | 5907.6 | 12.26% | motif file (matrix) | svg |
| 29 | T G C A A G C T A C G T C T A G G A T C C T A G G A T C G T C A C T G A A G T C | CEBP(bZIP)/ThioMac-CEBPb-ChIP-Seq(GSE21512)/Homer | 1e-7 | -1.680e+01 | 0.0000 | 181.0 | 18.10% | 5883.3 | 12.21% | motif file (matrix) | svg |
| 30 | G T A C G A T C C A G T A G T C A G T C A G T C T G C A G A T C C T G A A T G C G T C A A C G T | WT1(Zf)/Kidney-WT1-ChIP-Seq(GSE90016)/Homer | 1e-6 | -1.608e+01 | 0.0000 | 77.0 | 7.70% | 1949.7 | 4.05% | motif file (matrix) | svg |
| 31 | G T A C G C A T C T G A C G T A G A C T A G C T C A T G T G C A C T G A A C G T G A C T C G T A | Prop1(Homeobox)/GHFT1-PROP1.biotin-ChIP-Seq(GSE77302)/Homer | 1e-6 | -1.574e+01 | 0.0000 | 237.0 | 23.70% | 8315.6 | 17.26% | motif file (matrix) | svg |
| 32 | A C T G C T A G T C G A C G A T C A T G G C T A A T C G C G A T G T A C G C T A A G C T G T A C | Fra1(bZIP)/BT549-Fra1-ChIP-Seq(GSE46166)/Homer | 1e-6 | -1.441e+01 | 0.0000 | 122.0 | 12.20% | 3725.3 | 7.73% | motif file (matrix) | svg |
| 33 | C A G T T G C A A C G T A C T G C G T A A T C G C G A T T G A C C G T A A C G T | BATF(bZIP)/Th17-BATF-ChIP-Seq(GSE39756)/Homer | 1e-6 | -1.431e+01 | 0.0000 | 141.0 | 14.10% | 4482.1 | 9.31% | motif file (matrix) | svg |
| 34 | C T A G T C G A G C A T C A T G G C T A T A G C C G A T G T A C C T G A A G C T | JunB(bZIP)/DendriticCells-Junb-ChIP-Seq(GSE36099)/Homer | 1e-6 | -1.382e+01 | 0.0000 | 116.0 | 11.60% | 3536.6 | 7.34% | motif file (matrix) | svg |
| 35 | T A G C G A T C G T A C C T G A G C A T C T G A C G T A T G C A C G T A G A T C | Hoxa13(Homeobox)/ChickenMSG-Hoxa13.Flag-ChIP-Seq(GSE86088)/Homer | 1e-5 | -1.357e+01 | 0.0000 | 621.0 | 62.10% | 26343.8 | 54.69% | motif file (matrix) | svg |
| 36 | G A C T C G A T C T G A G T C A G A C T C G A T T C G A C G T A G C T A G C T A T G A C G T A C C G T A A C T G T G C A C G A T A C T G A C G T | Pitx1:Ebox(Homeobox,bHLH)/Hindlimb-Pitx1-ChIP-Seq(GSE41591)/Homer | 1e-5 | -1.323e+01 | 0.0000 | 71.0 | 7.10% | 1886.3 | 3.92% | motif file (matrix) | svg |
| 37 | T C G A A C G T C A T G G C T A T A G C C G A T G T A C G C T A A C G T A T G C | AP-1(bZIP)/ThioMac-PU.1-ChIP-Seq(GSE21512)/Homer | 1e-5 | -1.300e+01 | 0.0000 | 145.0 | 14.50% | 4755.8 | 9.87% | motif file (matrix) | svg |
| 38 | T C G A A G T C C G T A A T C G T A G C A C G T A C T G A G C T A C G T A G T C | Ptf1a(bHLH)/Panc1-Ptf1a-ChIP-Seq(GSE47459)/Homer | 1e-5 | -1.251e+01 | 0.0000 | 362.0 | 36.20% | 14239.0 | 29.56% | motif file (matrix) | svg |
| 39 | A C T G C A T G T C G A A C G T A C T G C G T A A T C G A C G T G T A C C G T A G A C T A G T C | Fos(bZIP)/TSC-Fos-ChIP-Seq(GSE110950)/Homer | 1e-5 | -1.251e+01 | 0.0000 | 125.0 | 12.50% | 3994.5 | 8.29% | motif file (matrix) | svg |
| 40 | C T A G T A C G G A T C G T C A T G C A A C G T T G C A G C T A T C G A T G C A | Hoxa9(Homeobox)/ChickenMSG-Hoxa9.Flag-ChIP-Seq(GSE86088)/Homer | 1e-5 | -1.208e+01 | 0.0001 | 591.0 | 59.10% | 25113.4 | 52.14% | motif file (matrix) | svg |
| 41 | A G C T A C G T A C T G A T G C A G T C C G T A C T G A T A C G | NF1-halfsite(CTF)/LNCaP-NF1-ChIP-Seq(Unpublished)/Homer | 1e-5 | -1.205e+01 | 0.0001 | 252.0 | 25.20% | 9385.1 | 19.48% | motif file (matrix) | svg |
| 42 | C A T G C T A G T C G A A C G T A C T G C G T A T A G C C G A T T G A C C G T A A G C T G A T C | Fra2(bZIP)/Striatum-Fra2-ChIP-Seq(GSE43429)/Homer | 1e-5 | -1.186e+01 | 0.0001 | 95.0 | 9.50% | 2868.5 | 5.96% | motif file (matrix) | svg |
| 43 | G A T C G C A T C T G A C G T A A G C T A G C T C G T A T C G A C T G A A C G T G C A T C G T A | Phox2a(Homeobox)/Neuron-Phox2a-ChIP-Seq(GSE31456)/Homer | 1e-4 | -1.138e+01 | 0.0001 | 150.0 | 15.00% | 5113.1 | 10.62% | motif file (matrix) | svg |
| 44 | G A C T G T A C T G C A A C G T G A T C G C T A T C G A A C G T A G T C C G T A | Pdx1(Homeobox)/Islet-Pdx1-ChIP-Seq(SRA008281)/Homer | 1e-4 | -1.103e+01 | 0.0002 | 246.0 | 24.60% | 9255.4 | 19.22% | motif file (matrix) | svg |
| 45 | C T A G A G T C T A C G T A C G T G A C C G T A A C T G T A G C G C A T C A T G A T G C A G C T | Ascl1(bHLH)/NeuralTubes-Ascl1-ChIP-Seq(GSE55840)/Homer | 1e-4 | -1.072e+01 | 0.0002 | 198.0 | 19.80% | 7211.0 | 14.97% | motif file (matrix) | svg |
| 46 | T C A G T A C G G A T C G T A C T C G A C G A T C T G A C G T A G C T A C G T A | Hoxd11(Homeobox)/ChickenMSG-Hoxd11.Flag-ChIP-Seq(GSE86088)/Homer | 1e-4 | -1.058e+01 | 0.0002 | 568.0 | 56.80% | 24254.1 | 50.36% | motif file (matrix) | svg |
| 47 | C A T G A C T G C T A G T C G A T C G A T C G A T C G A T C A G T C A G T C A G T G A C T G A C C G T A A C T G T G C A C G A T A C T G | RBPJ:Ebox(?,bHLH)/Panc1-Rbpj1-ChIP-Seq(GSE47459)/Homer | 1e-4 | -1.056e+01 | 0.0002 | 61.0 | 6.10% | 1677.8 | 3.48% | motif file (matrix) | svg |
| 48 | A T G C G A C T A C T G C A G T G A T C A C G T T A C G T A C G | Smad2(MAD)/ES-SMAD2-ChIP-Seq(GSE29422)/Homer | 1e-4 | -1.053e+01 | 0.0002 | 225.0 | 22.50% | 8403.2 | 17.45% | motif file (matrix) | svg |
| 49 | G C A T C G T A C G A T A C T G A G T C G C T A C T G A C G T A C A G T A C T G C G T A T C A G | Oct6(POU,Homeobox)/NPC-Pou3f1-ChIP-Seq(GSE35496)/Homer | 1e-4 | -9.971e+00 | 0.0004 | 142.0 | 14.20% | 4926.8 | 10.23% | motif file (matrix) | svg |
| 50 | C G T A T A G C A G T C C T A G C A G T C T A G C T G A G T A C G C A T T C G A C G T A G C A T A G C T C T A G T C G A | PAX3:FKHR-fusion(Paired,Homeobox)/Rh4-PAX3:FKHR-ChIP-Seq(GSE19063)/Homer | 1e-3 | -8.863e+00 | 0.0012 | 69.0 | 6.90% | 2089.9 | 4.34% | motif file (matrix) | svg |
| 51 | G A C T C G T A A C G T A C T G A G T C C G T A C T G A C G T A C A G T A C G T G T C A T C A G | Brn1(POU,Homeobox)/NPC-Brn1-ChIP-Seq(GSE35496)/Homer | 1e-3 | -8.834e+00 | 0.0013 | 104.0 | 10.40% | 3478.3 | 7.22% | motif file (matrix) | svg |
| 52 | C A G T C G A T G C A T G C A T G T C A A G C T C A T G C T A G A T G C G T A C | Hoxa11(Homeobox)/ChickenMSG-Hoxa11.Flag-ChIP-Seq(GSE86088)/Homer | 1e-3 | -8.489e+00 | 0.0017 | 565.0 | 56.50% | 24504.7 | 50.88% | motif file (matrix) | svg |
| 53 | T G A C A T G C C G T A A T C G A T G C C A G T C A T G A C T G A G T C G T A C | HEB(bHLH)/mES-Heb-ChIP-Seq(GSE53233)/Homer | 1e-3 | -7.975e+00 | 0.0029 | 231.0 | 23.10% | 9034.8 | 18.76% | motif file (matrix) | svg |
| 54 | C T A G T C G A C G A T A C T G C G T A T A C G A G C T T G A C G C T A A C G T G A T C T A G C | Fosl2(bZIP)/3T3L1-Fosl2-ChIP-Seq(GSE56872)/Homer | 1e-3 | -7.894e+00 | 0.0030 | 59.0 | 5.90% | 1777.4 | 3.69% | motif file (matrix) | svg |
| 55 | G A C T G A C T T C G A C G T A C G A T A G C T C T G A A C T G T G A C G A C T T C G A C G T A A C G T A G C T C T G A C T G A G T C A G C T A G C T A C G T A | Pax7(Paired,Homeobox),longest/Myoblast-Pax7-ChIP-Seq(GSE25064)/Homer | 1e-3 | -7.434e+00 | 0.0047 | 22.0 | 2.20% | 478.0 | 0.99% | motif file (matrix) | svg |
| 56 | C A G T T C A G A G C T G A C T A C G T A G T C G A T C G A C T C T G A A C T G G A T C C G T A C T G A A G T C G T A C | Rfx6(HTH)/Min6b1-Rfx6.HA-ChIP-Seq(GSE62844)/Homer | 1e-3 | -7.406e+00 | 0.0048 | 157.0 | 15.70% | 5871.1 | 12.19% | motif file (matrix) | svg |
| 57 | T G C A T C G A T C G A A C G T C A T G C G T A A G T C T C A G A C G T G T A C C G T A A G C T | CREB5(bZIP)/LNCaP-CREB5.V5-ChIP-Seq(GSE137775)/Homer | 1e-3 | -7.058e+00 | 0.0066 | 78.0 | 7.80% | 2595.8 | 5.39% | motif file (matrix) | svg |
| 58 | C T A G T A C G G A C T T G C A T G C A C G A T T A C G C T G A T C G A C T G A | Hoxa10(Homeobox)/ChickenMSG-Hoxa10.Flag-ChIP-Seq(GSE86088)/Homer | 1e-2 | -6.573e+00 | 0.0106 | 154.0 | 15.40% | 5859.4 | 12.17% | motif file (matrix) | svg |
| 59 | T A C G A T C G T A G C G A T C A C T G A C G T A G T C A C G T C T A G A T C G | Smad4(MAD)/ESC-SMAD4-ChIP-Seq(GSE29422)/Homer | 1e-2 | -6.546e+00 | 0.0107 | 212.0 | 21.20% | 8423.1 | 17.49% | motif file (matrix) | svg |
| 60 | T A G C G T A C C G T A C T A G A C T G T G C A C G T A A T G C C G T A A T C G | AR-halfsite(NR)/LNCaP-AR-ChIP-Seq(GSE27824)/Homer | 1e-2 | -6.455e+00 | 0.0115 | 556.0 | 55.60% | 24510.8 | 50.89% | motif file (matrix) | svg |
| 61 | T G C A A G C T C A T G C G T A A G C T A C T G G A T C G T C A C G T A A G C T | Atf4(bZIP)/MEF-Atf4-ChIP-Seq(GSE35681)/Homer | 1e-2 | -6.311e+00 | 0.0131 | 66.0 | 6.60% | 2182.9 | 4.53% | motif file (matrix) | svg |
| 62 | G C T A G A C T G A C T T G C A C G T A A G T C C G T A T A G C G A T C G A C T | Eomes(T-box)/H9-Eomes-ChIP-Seq(GSE26097)/Homer | 1e-2 | -5.902e+00 | 0.0194 | 378.0 | 37.80% | 16168.5 | 33.57% | motif file (matrix) | svg |
| 63 | C G A T C T G A C G T A A G C T A G T C T G C A C T G A A C G T A G C T G C T A | Pax7(Paired,Homeobox)/Myoblast-Pax7-ChIP-Seq(GSE25064)/Homer | 1e-2 | -5.835e+00 | 0.0204 | 41.0 | 4.10% | 1239.9 | 2.57% | motif file (matrix) | svg |
| 64 | C G T A C T G A C T A G C G T A C G T A A G T C C G T A C A G T G C A T G T C A C G A T A C T G A C G T G C A T G A T C | PGR(NR)/EndoStromal-PGR-ChIP-Seq(GSE69539)/Homer | 1e-2 | -5.501e+00 | 0.0281 | 60.0 | 6.00% | 2017.3 | 4.19% | motif file (matrix) | svg |
| 65 | C T A G T C G A A C G T A C T G C G T A T A G C C G A T G T A C C G T A A G C T G A T C G T A C | Jun-AP1(bZIP)/K562-cJun-ChIP-Seq(GSE31477)/Homer | 1e-2 | -5.373e+00 | 0.0314 | 40.0 | 4.00% | 1235.7 | 2.57% | motif file (matrix) | svg |
| 66 | G A C T C T A G C T A G A G T C T G C A A C T G A C G T A C G T C T A G T C A G | AMYB(HTH)/Testes-AMYB-ChIP-Seq(GSE44588)/Homer | 1e-2 | -5.361e+00 | 0.0314 | 251.0 | 25.10% | 10412.5 | 21.62% | motif file (matrix) | svg |
| 67 | C A G T T C A G G A T C A C T G A C G T C T A G A C T G A C T G G A C T C T A G | Egr1(Zf)/K562-Egr1-ChIP-Seq(GSE32465)/Homer | 1e-2 | -5.361e+00 | 0.0314 | 63.0 | 6.30% | 2153.8 | 4.47% | motif file (matrix) | svg |
| 68 | A G T C G T A C A G C T C T A G A G T C C G A T A C T G C G T A A C T G G T C A | Zic(Zf)/Cerebellum-ZIC1.2-ChIP-Seq(GSE60731)/Homer | 1e-2 | -5.325e+00 | 0.0315 | 106.0 | 10.60% | 3963.6 | 8.23% | motif file (matrix) | svg |
| 69 | C G T A A C G T A C G T A C G T A C G T A G T C A G T C C T G A A G C T A G C T | NFAT(RHD)/Jurkat-NFATC1-ChIP-Seq(Jolma\_et\_al.)/Homer | 1e-2 | -5.268e+00 | 0.0329 | 197.0 | 19.70% | 7983.1 | 16.57% | motif file (matrix) | svg |
| 70 | A C T G A C G T C A T G A T C G A T C G T G A C A C T G A T C G A T C G T G C A C T G A C G T A | E2F3(E2F)/MEF-E2F3-ChIP-Seq(GSE71376)/Homer | 1e-2 | -5.239e+00 | 0.0334 | 38.0 | 3.80% | 1168.4 | 2.43% | motif file (matrix) | svg |
| 71 | C T G A T C A G C A G T C T A G A C T G C T A G G A T C A T C G A C T G C T G A T C A G G A T C | Sp5(Zf)/mES-Sp5.Flag-ChIP-Seq(GSE72989)/Homer | 1e-2 | -5.156e+00 | 0.0357 | 70.0 | 7.00% | 2463.6 | 5.11% | motif file (matrix) | svg |
| 72 | T A C G C T A G T A C G A G T C C G T A A G T C A G T C A C G T A C T G A G T C G A T C T A G C | Slug(Zf)/Mesoderm-Snai2-ChIP-Seq(GSE61475)/Homer | 1e-2 | -4.970e+00 | 0.0425 | 68.0 | 6.80% | 2401.1 | 4.98% | motif file (matrix) | svg |
| 73 | A G C T C A T G G C A T G A T C T G C A C T A G G A T C A C G T | Tgif2(Homeobox)/mES-Tgif2-ChIP-Seq(GSE55404)/Homer | 1e-2 | -4.849e+00 | 0.0472 | 497.0 | 49.70% | 22078.0 | 45.84% | motif file (matrix) | svg |
| 74 | C T A G T C G A C G A T C T A G G C A T C A G T C T A G G A T C C G T A G T C A | CEBP:AP1(bZIP)/ThioMac-CEBPb-ChIP-Seq(GSE21512)/Homer | 1e-2 | -4.832e+00 | 0.0474 | 153.0 | 15.30% | 6097.9 | 12.66% | motif file (matrix) | svg |
| 75 | A G T C A C G T A C T G A G C T A C G T A C G T G T C A A G T C | Foxo1(Forkhead)/RAW-Foxo1-ChIP-Seq(Fan\_et\_al.)/Homer | 1e-2 | -4.825e+00 | 0.0474 | 333.0 | 33.30% | 14324.3 | 29.74% | motif file (matrix) | svg |
